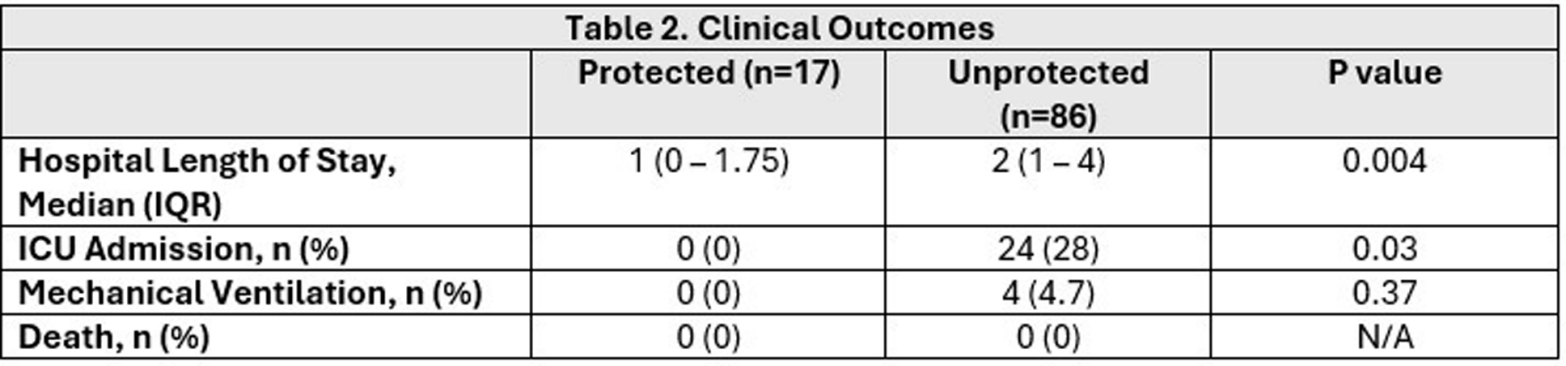# 336 Appropriateness of urinalysis bypass and effect on antibiotic prescribing for urinary tract infections in the outpatient setting

**DOI:** 10.1017/ash.2026.10454

**Published:** 2026-06-23

**Authors:** Donna Wesslen, Eileen Campbell, Julie Williamson, Wyn Wheeler, Rupal Jaffa, Catherine Passaretti, Anupama Neelakanta

**Affiliations:** 1 Atrium Health; 2 Division of Pharmacy, Antimicrobial Support Network, Atrium Health; 3 Advocate Health

## Abstract

**Background:** Respiratory Syncytial Virus (RSV) is a leading cause of infant hospitalizations in the United States and is particularly devastating to infants 6 months and younger. Maternal RSV vaccination given more than two weeks before delivery and early infant nirsevimab administration can provide protection against severe illness in this vulnerable population. Objective: To evaluate uptake of, and barriers to, RSV immunization among infants admitted to the hospital with RSV infection in a large healthcare system. Additionally, clinical outcomes between infants who did and did not receive any RSV protection were compared. **Methods:** This was a retrospective cohort of infants ? 6 months old hospitalized in a large multi-state healthcare system from November 2024 to January 2025 with a positive RSV test within 7 days prior to or during admission. Patients were identified using an electronic health record report and chart review was performed to obtain data on RSV immunization and clinical outcomes. Infants born outside of our healthcare system where information on RSV immunization was lacking were excluded. Infants were classified as protected if either 1) maternal RSV vaccination was given more than two weeks before delivery or 2) the infant received nirsevimab prior to the onset of RSV infection. All others were classified as unprotected. Clinical outcomes including length of stay, ICU admission, and mechanical ventilation were compared between groups. Identification of barriers to RSV immunization were attempted. **Result:** Of 152 infants admitted with RSV, 103 were born in our hospitals and included. 17 (16.5%) patients were considered protected (8 with maternal vaccination; 9 infant nirsevimab administrations). Patients in both groups were predominantly male and either Non-Hispanic White or Hispanic. Compared to unprotected patients, protected patients had significantly fewer ICU admissions (0% vs 28%, p = 0.03) and shorter median length of stay (1 vs 2 days, p=0.004). There was no difference between groups in rates of mechanical ventilation or death. Among unprotected patients, chart review most often showed no documentation of any RSV immunization discussions (75.6%), family declined treatment (12.8%), and infants becoming ill before nirsevimab could be administered despite willingness to eventually receive it (11.6%). Post-RSV infection, an additional 12% of unprotected patients received nirsevimab. **Conclusion:** Unprotected infants were more likely to experience negative clinical outcomes, specifically more ICU admissions and longer hospitalizations. Opportunities exist to improve RSV provider and family education and implement interventions to facilitate discussion and administration of RSV prophylaxis.

**Figure:**
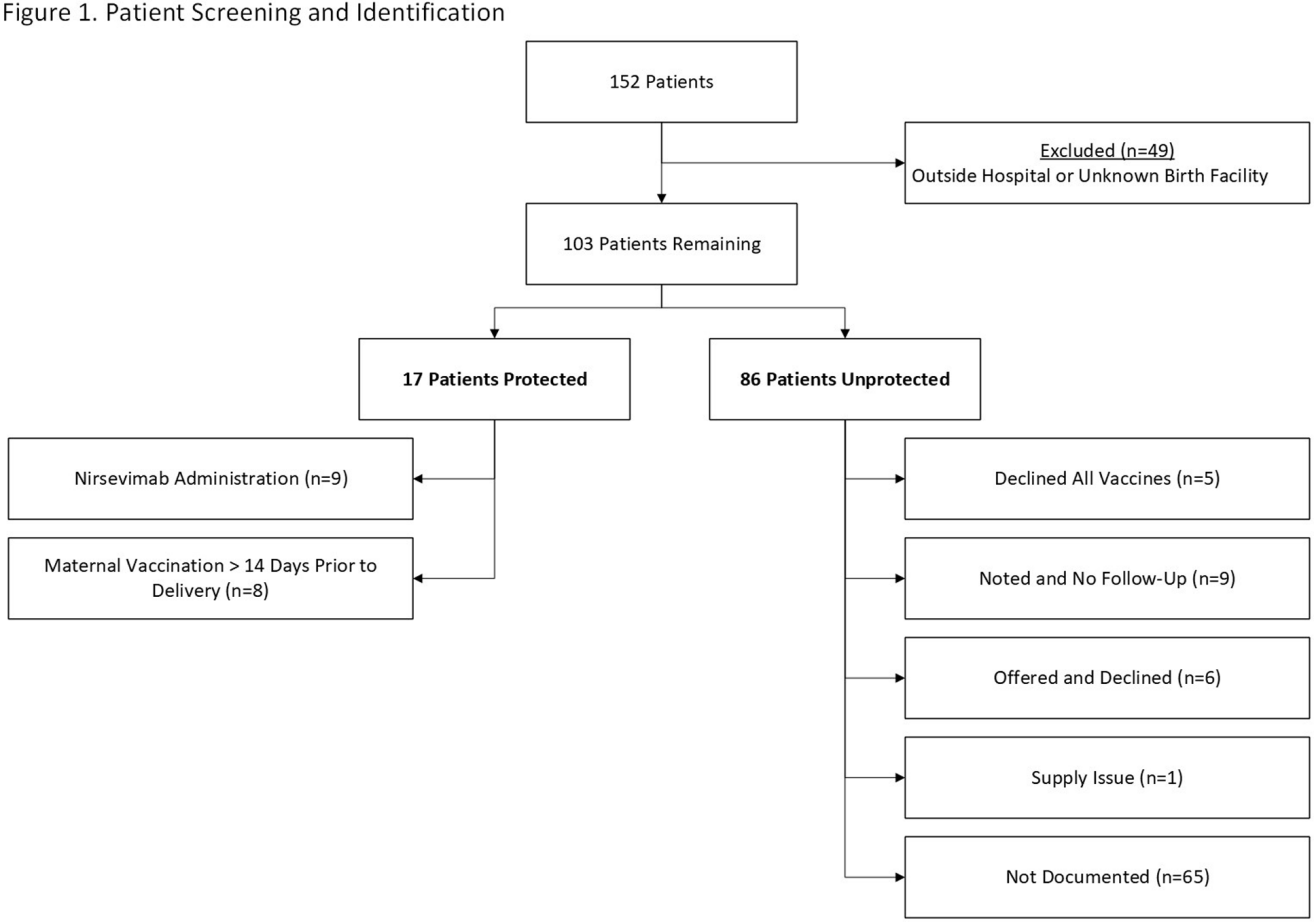

**Figure:**
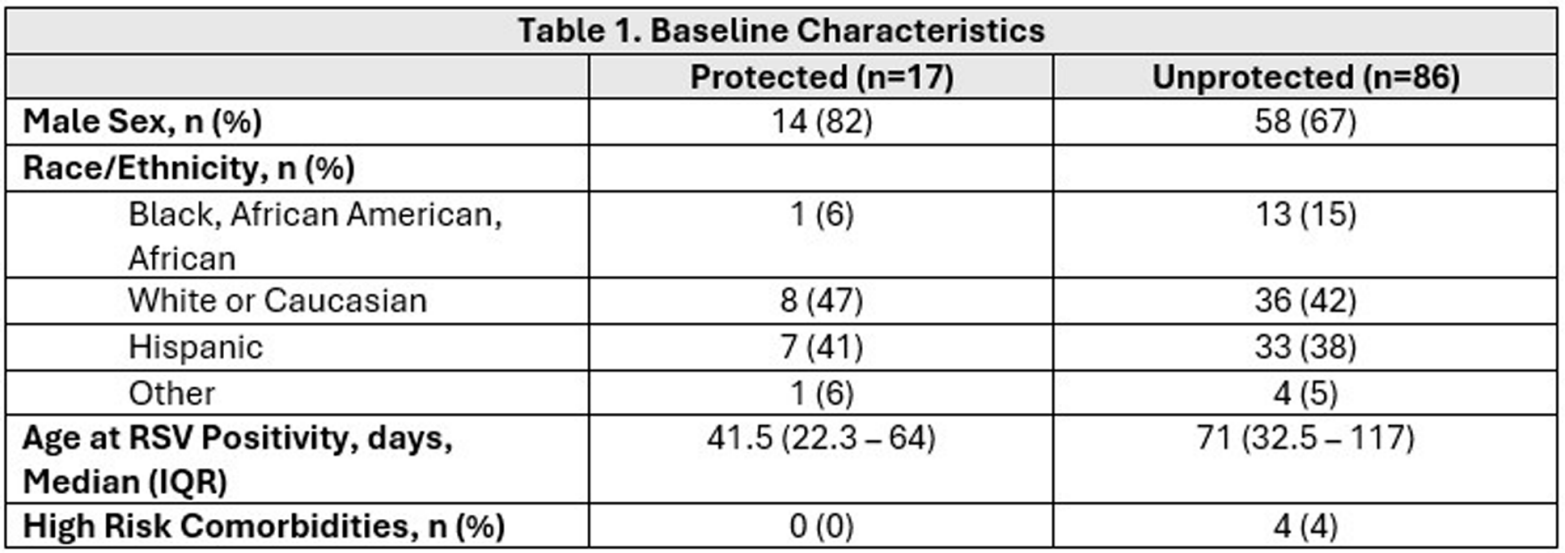

**Figure:**